# Division of Responsibility in Child Feeding and Eating Competence: A Cross-Sectional Study in a Sample of Caregivers of Brazilian Children with Celiac Disease

**DOI:** 10.3390/nu16071052

**Published:** 2024-04-04

**Authors:** Larissa Caetano Silva, Eduardo Yoshio Nakano, Renata Puppin Zandonadi

**Affiliations:** 1 Nutrition Department, Faculty of Health Sciences, University of Brasília, Campus Universitário Darcy Ribeiro, Brasília 70910-900, Brazil; larissacaetanos@gmail.com; 2 Department of Statistics, University of Brasília, Campus Universitário Darcy Ribeiro, Brasília 70910-900, Brazil; nakano@unb.br

**Keywords:** infant feeding, division of responsibility in feeding, eating competence, Brazilian children, caregivers, celiac disease, gluten-free, eating behavior

## Abstract

The objective of this cross-sectional study was to assess eating competence (EC) and the adherence to the division of responsibility in child feeding (sDOR) of Brazilian caregivers of children with celiac disease (CD). It also examined the association between EC and sDOR, children’s adherence to a gluten-free diet, and sociodemographic data. This study administered a survey set that included sociodemographic data, health-related data, eating habits, and the instruments ecSI2.0^TM^BR and sDOR.2-6y^TM^ BR, validated for a Brazilian population. The sample comprised 50 caregivers of children with CD (between 24 and 72 months of age). The participants following a gluten-free diet (GFD) presented higher scores for all EC domains and the total EC. The total EC scores were higher for the participants over 40 y/o, frequently having meals as a family, with their children consuming more than three servings of fruit and at least one serving of vegetables daily and complying with a GFD. Different from the EC, the sDOR.2-6y^TM^ scores did not differ between the participants complying with a GFD. The sDOR.2-6y^TM^ mealtime structure domain scores were significantly associated with the EC eating attitude, food acceptance, contextual skills, and total. These findings support the need for greater attention to exploring the division of responsibility in feeding and EC in pediatric celiac disease, potentially enhancing intervention strategies for patients and their families.

## 1. Introduction

Children’s feeding is a reciprocal process, with the interaction between the child’s signs of hunger and satiety, the caregiver’s responsiveness to these signs, and the effect that this has on the child’s eating self-regulation [1]. Children’s feeding depends on the skills of both caregivers and children, who are in a didactic relationship in which eating practices which allow children to eat autonomously should be implemented, stimulating eating self-regulation and supporting cognitive, emotional, and social development [2,3].

In this context, the division of responsibility in child feeding (sDOR) defines that caregivers are responsible for deciding what, when, and where food will be offered, while children are responsible for determining what and in what quantity food will be consumed [4,5,6]. Another concept involved in feeding is eating competence (EC), which is described as an attitudinal and behavioral model toward eating which has been associated with a greater diet quality and better nutrition, considering four components: eating attitude, food acceptability, internal regulation of physical signs of hunger and satiety, and contextual skills, linked to managing one’s diet [7]. Considering the two models, parents who are competent eaters tend to implement more positive eating practices in their children’s diet and follow more of the principles described in the sDOR. Furthermore, by following the principles of the sDOR the child is expected to become a competent eater [6,8].

In diseases with a dietary treatment, such as celiac disease (CD), clarifying food choices and eating behaviors can promote compliance with the restricted diet and adapt interventions. CD is a chronic immune-mediated systemic disease provoked by gluten (from wheat, barley, rye) ingestion in susceptible individuals. Celiac disease is considered a public health problem and affects about 1% of the worldwide pediatric population [9]. Despite the considerable increase in its prevalence in the last 50 years, this disease remains with high estimates of underdiagnosis [10], probably due to its broad clinical spectrum [11]. To date, the only safe treatment is a lifelong gluten-free diet (GFD) [12].

Parents’ and caregivers’ engagement in maintaining an age-appropriate diet and adhering to dietary restrictions deriving from CD is of utmost importance [13], since strict GFD adherence is challenging and involves knowledge about the diet and gluten-free products, labeling, food safety, social repercussions, and the cost and availability of gluten-free foods [11,14,15,16,17,18,19]. In addition, another concern with a GFD is keeping it nutritionally balanced, providing adequate growth and development in children [20]. A recent study in Brazil evaluated the division of responsibility in infant feeding and EC among Brazilian caregivers in general (n = 549) [21]. Other Brazilian studies have been developed applying the ecSI2.0^TM^BR in a sample of 1030 adults diagnosed with gluten-related disorders, most of them being celiac [22], and the general population (n = 1810) [23]. However, no study has assessed the association between eating competence, the division of responsibility in feeding, and children with CD’s adherence to the gluten-free diet in Brazil.

In this sense, it was hypothesized that the parents or caregivers of a child with gluten-related disorders have high scores in terms of eating competence and lower scores in the division of responsibility in feeding. The secondary hypothesis is that the caregivers of children with CD who comply with a GFD have higher ecSI 2.0^TM^ and sDOR.2-6y^TM^ scores than those whose children do not comply with a GFD. Therefore, this study aimed to assess the association between eating competence, the division of responsibility in feeding, children’s adherence to the gluten-free diet, and the sociodemographic data of the participants.

## 2. Materials and Methods

### 2.1. Study Design, Sample, and Ethical Aspects

This cross-sectional study was conducted with a convenience sampling method using the online snowball enrollment of caregivers of children with CD aged between 24 and 72 months. This study was approved by the Research Ethics Committee (CAAE 56301222.1.0000.0030), and the NEEDs Center [24] authorized the use of the instruments ecSI2.0^TM^BR and sDOR.2-6y^TM^ BR.

The inclusion criteria were as follows: (i) being a Brazilian adult (≥19 years old); (ii) being a parent or caregiver of a CD child aged between 24 and 72 months; and (iii) accepting to participate in this study after having read and agreed through the informed consent form. The exclusion criteria for participating in this study were the following: (i) individuals who did not consent to their involvement; and (ii) individuals who did not complete all the parts of the survey.

### 2.2. Instrument Application

The survey comprised four parts: (i) Caregivers’ sociodemographic data. (ii) Satter Eating Competence Inventory version validated for the Brazilian population (ecSI2.0^TM^BR). The ecSI2.0^TM^BR is a sixteen-item inventory divided into four components: eating attitude, related to being positive and calm about food and eating; food acceptance, related to interest in food and consuming varied and new foods; internal regulation, related to attention to signs of hunger and satiety; and contextual skills, related to the management of food and food context [7]. Each item has five response options (always, often, sometimes, rarely, and never), scored from 3 to 0, so that the possible total scores range from 0 to 48, with higher numbers indicating a greater EC [6,7]. The ecSI2.0^TM^ classifies individuals into competent (ecSI2.0^TM^ ≥ 32) and non-competent eaters [25]. (iii) Satter Division of Responsibility in Feeding version validated for the Brazilian population (sDOR.2-6y^TM^ BR). The sDOR.2-6y^TM^BR is a questionnaire composed of 12 items, whose domains are mealtime structure (D1), what is available to the child (D2), how food is available to the child (D3), respect for the child’s autonomy in eating (D4), and who controls what, when, and how much is eaten (D5). Each item has five response options (always, often, sometimes, rarely, and never), scored from 3 to 0 [6]. The total score varies from 0 to 36; the higher the score, the higher the parents’ adherence to the sDOR.2-6y^TM^BR. Scores above 24 represent a good adherence to the sDOR.2-6y^TM^BR. The sDOR.2-6y^TM^ Portuguese—Brazil and ecSI2.0^TM^BR were validated to be applied to the Brazilian population [6,8,21,26,27,28]. (iv) Questions about children’s GFD compliance, health, and feeding (how long has the child been diagnosed with celiac disease; whether the child has any other medical diagnosis in addition to CD; if the child complies with a GFD; weekly frequency of family meals; weekly frequency of homemade meals; fruit and vegetables’ daily consumption; if they received nutritional guidance at school, in clinics/offices, or another health service in the last 12 months; and if they are part of celiac disease groups).

The caregivers’ GFD compliance was self-reported, since there is no validated instrument to evaluate GFD compliance in Brazil. Therefore, self-reported GFD compliance was used following other studies [19,22,29,30,31,32,33]. The participants chose the option that best characterized their current diet regarding the following question: “Do you comply with a gluten-free diet?”. The response options were as follows: (1) never; (2) rarely; (3) sometimes; (4) almost always (most of the time); and (5) always. Strict GFD compliance was considered for those who self-reported always adhering to a GFD, whereas all others were considered to be “gluten-exposed”. All the participants filled out both questionnaires. The question about children’s GFD compliance (“Does your child comply with GFD?”) was reported by the parents following the same method. The amount of fruits and vegetables consumed by the child on a typical day was reported by the parents (“How many servings of fruit does your child eat on a typical day?”—response options: (1) none; (2) 1–2 servings per day; (3) 3–4 servings per day; and (4) 5 or more servings per day; “How many servings of vegetables does your child eat on a typical day?”—response options: (1) none; (2) 1–3 servings per day; (3) 4–6 servings per day; and (4) 7 or more servings per day).

The instrument was inserted into the Google Forms© tool and spread via social media from April to September 2023. The research reached coverage in all Brazilian regions. Data collection occurred through non-probabilistic convenience sampling by virtual recruitment using the “snowball” method [34]. This method was selected due to Brazil’s size, making it difficult for in-person collection. Moreover, “snowball” sampling spread via social media is considered an effective and efficient method of recruiting participants that allows to achieve a larger sample size, with a shorter completion time and a low application cost [34,35]. All the tool responses were scored according to the guidelines published in the original research [36,37].

The participants’ recruitment occurred by different strategies with the support of Brazilian celiac entities (Brazilian Celiac Associations, ACELBRAs; National Federation of Celiac Associations in Brazil, FENACELBRA) as well as food service websites, stores, and restaurants that serve gluten-free foods or personal webpages offering posts about gluten-related disorders, and invitation for this study was disseminated using social media (emails, Facebook groups, WhatsApp, and Instagram). People who accessed the research link were invited to share it with acquaintances who fit the target audience.

### 2.3. Statistical Analysis

IBM SPSS (IBM SPSS Statistics for Windows, IBM Corp., Armonk, NY, USA) version 22 was used for the analysis. The participant characteristics were reported using descriptive statistics. The ecSI2.0™BR and sDOR.2-6y^TM^BR scores were described by means and standard deviations (SD). Student’s *t*-test and an analysis of variance (ANOVA), followed by Tukey’s post hoc tests, were used to compare the ecSI2.0™BR and sDOR.2-6y^TM^BR scores with the variables of interest. The Kolmogorov–Smirnov test verified the normality assumption. The association between the sDOR.2-6y^TM^ BR scores and the ecSI2.0^TM^BR scores was verified by means of Pearson’s chi-squared and Fisher’s exact test. The Cronbach’s alpha coefficient was calculated to analyze the internal consistency of ecSI2.0™BR and sDOR.2-6y^TM^BR. All the tests were performed considering bilateral hypotheses and a 5% significance level.

## 3. Results

### 3.1. Sociodemographic Data

Our sample comprised 50 participants, primarily females (n = 49; 98%), up to 40 y/o (n = 39; 78%), some of them graduates (n = 27; 54%), with an income up to five times the minimum wage (n = 24; 48%), most of them of a normal weight (n = 26; 54.2%), and not complying with a GFD (n = 28; 56%), with mostly female children with CD (n = 32; 64%), some of whom had been diagnosed with CD for less than one year (n = 22; 44%) and had no other medical diagnosis besides CD (n = 28; 56%). Most of the children complied with a GFD (n = 41; 82%), having meals with their family (n = 44; 88%) which were frequently prepared at home (n = 48; 96%), guided by a dietitian (n = 28; 56%), and participating in CD groups/associations (n = 42; 84%). Most of the children consumed 1–2 servings of fruits (n = 34; 68%) and 1–2 servings of vegetables (n = 36; 72%) daily (Table S1—Supplementary File).

### 3.2. ecSI2.0^TM^BR

The mean score of the ecSI2.0^TM^BR in this study was 31.98 ± 9.28. The participants following a GFD presented higher scores for all the ecSI2.0^TM^BR domains and the total. The ecSI2.0^TM^BR presented a good internal consistency. The Cronbach’s alpha coefficient for the total score was 0.891. Also, the eating attitude domain’s scores were higher for the participants complying with a GFD whose children were 5–6 y/o and consumed vegetables at least 1×/day. The food acceptance scores were higher for the participants frequently having meals as a family, with children consuming more than three servings of fruit and at least one serving of vegetables daily. The internal regulation scores were higher for the participants whose children were 5–6 y/o, complied with a GFD, and consumed more than three servings of fruit and at least one serving of vegetables daily. The contextual skills’ scores were higher for those over 40 y/o, frequently having meals as a family, with children consuming at least one serving of vegetables daily and complying with a GFD. The total EC scores were higher for the participants who were more than 40 y/o, frequently having meals as a family, with children consuming more than three servings of fruit and at least one serving of vegetables daily and complying with a GFD (Table 1).

### 3.3. sDOR.2-6y^TM^BR

The mean score of the sDOR.2-6y^TM^BR in this study was 22.08 ± 3.86. The Cronbach’s alpha coefficient for the total score of the sDOR.2-6y^TM^BR was 0.221. Although this sample showed a low Cronbach’s alpha value, this value was close to that found in another Brazilian study on the sDOR.2-6y^TM^BR (Cronbach’s alpha = 0.301) [21,38]. Table 2 shows the sub-scores and categories of the sDOR.2-6y^TM^BR scales categorized by sociodemographic variables and health and consumption characteristics. Different from the EC, the sDOR.2-6y^TM^BR scores did not differ among the participants complying with a GFD. The participants whose children complied with a GFD had higher scores for D1 (mealtime structure) but lower scores for D2 (what is available to the child). The income differed only for D3 (how food is available to the child), in which those participants with up to 2 MW and >10 MW presented the best scores. The participants frequently having meals with their family presented the best scores for D1 (mealtime structure) and D3 (how food is available to the child). The participants whose children had been diagnosed with CD for less than one year presented higher scores for D5 (how much is eaten), and those with children with CD diagnoses more than 3 years prior to this study showed the best scores for the same domain.

### 3.4. Associations between sDOR.2-6y^TM^ and ecSI2.0^TM^BR

Table 3 shows that there are associations only for sDOR.2-6y^TM^ D1 and EC’s eating attitude, food acceptance, contextual skills, and total EC, considering the data received by Brazilian caregivers of children with celiac disease.

## 4. Discussion

This study evaluated the eating competence and the division of responsibility in feeding among Brazilian parents or caregivers of children with celiac disease aged 24–72 months. Despite the increasing role of fathers in taking responsibility for their children’s diet and their involvement in daily care [39,40], mothers are more concerned about their own health and their children’s health [39,40,41,42], especially in families with chronically ill children [43], which is reinforced by the female predominance of the sample in this study.

The participants complying with a GFD presented higher scores for all the ecSI2.0^TM^BR domains and the total score, meaning that they could still enjoy eating, be relaxed, and plan meals, confirming one of our hypotheses. A study with parents of school-age children living in the United States (n = 339; 78% Hispanic) showed that the group of parents with the highest ecSI2.0^TM^ scores demonstrated healthier eating behaviors, tried new foods, ate regular meals, set aside time to eat, and paid attention to their diet [8]. A GFD requires knowledge about food, management of the food environment, meal planning, and attention to labels, so a GFD likely needs one to meet the EC requirements. In a Brazilian study on individuals with gluten-related disorders (n = 1030), higher eating competence scores were found in adults with CD compared to the general population, with an association between compliance with a GFD and a higher total ecSI2.0^TM^BR score [22].

The mean score of the ecSI2.0^TM^BR in this study (31.98 ± 9.28) was lower than that of the study performed, in a more general context, among Brazilian parents or caregivers of children between 24 and 72 months of age (32.58 ± 7.75) [21].The total EC scores were higher for the participants over 40 y/o, frequently having meals as a family, with children consuming more than three servings of fruit and at least one serving of vegetables daily and complying with a GFD. The study carried out in Brazil among adults with gluten-related disorders showed that individuals classified as competent eaters (ecSI2.0^TM^ ≥ 32) mainly were over 40 y/o, complying with a GFD, and consuming homemade gluten-free products, prepared mainly by themselves [22]. Previous studies have also shown that parents with behaviors linked to cooking play an essential role in stimulating their children’s consumption of fruits and vegetables [8,44]. Two US studies evaluating the eating behavior and ecSI2.0^TM^ of parents of school-age children showed that the group of parents with the highest score on the ecSI2.0^TM^ had a higher frequency of consuming breakfast and dinner with their children, a greater availability of vegetables and fruits at home compared to the parents with lower scores on the ecSI2.0^TM^, in addition to exhibiting a greater frequency of preparing meals together with their children and a greater availability to cook [8,45]. Furthermore, EC is directly related to greater skills in food handling and managing one’s diet [46], which may explain the findings in this study.

EC is related to the highest consumption of fruits and vegetables [8] and the best adherence to the Mediterranean diet, considered indicators of healthy eating [47]. However, sometimes, parents dealing with their children’s food restriction tend to give in to their children’s food choices within what they can eat, not always making them have more appropriate choices but allowing the option for industrialized gluten-free products. A study in Italy with 120 children with celiac disease and 100 children without celiac disease showed that commercial gluten-free products specifically formulated for CD patients provided 46% of the total energy value required, influencing the imbalance in the diet of children with CD [20]. The same study, however, showed that neither group reached the number of servings recommended for legumes and vegetables, and the consumption of fruit was at the minimum recommended [20], which may represent a trend unrelated to CD.

A study assessing EC and its association with food selection, eating patterns, and related psychobehavioral factors in Finnish adolescents (n = 976), aged 10 to 17 years, showed an association between EC and a greater regularity of meals and a greater consumption of fruits and vegetables, in addition to healthier family eating patterns [48], similar to what was found in our study. A Korean cross-sectional study, with 363 mothers of children aged 2 to 5 years, also showed that fruit and vegetable consumption was positively influenced if the parents built a healthy home food environment [49].

The eating attitude domain scores, related to being positive about food and eating [25], were higher for the participants complying with a GFD, possibly because good adherence to the restricted diet keeps individuals calm about food choices. The food acceptance scores were higher for the participants frequently having meals as a family, with children consuming more than three servings of fruit and at least one serving of vegetables daily. Food acceptability is based on a positive interest in food, feeling calm in situations involving new foods, being able to make choices, accepting or refusing the foods offered as well as trying different foods [7], which may have negative repercussions due to previous experiences with adverse reactions to food in disease contexts. In our study, the controlled family environment may have brought security to the acceptability of foods. It is worth noting that individuals who are considered competent eaters tend to have a better diet quality and greater fiber, vitamin, and mineral intakes and make healthier choices [50].

The internal regulation scores were higher for the participants whose children were 5–6 y/o, complied with a GFD, and consumed more than three servings of fruit and at least one serving of vegetables daily. Internal regulation is related to the ability to identify physical signs of hunger, appetite, and satiety [7] and, in the case of parents, be attentive to the child’s signs of self-regulation. According to the approach proposed by Satter, it requires a balance between discipline, which includes having regular meals in an appropriate environment, and permission, which involves the possibility of being able to choose the foods for each meal and the freedom of being able to eat enough to feel satisfied [51,52]. Therefore, our findings do not allow us to determine the direction of the causal relationship: whether healthy choices and GFD compliance cause a greater child autonomy or whether a greater child autonomy causes better food choices.

The contextual skills scores were higher for those over 40 y/o, frequently having meals as a family, with children consuming at least one serving of vegetables daily and complying with a GFD. In the ecSatter model, this component is linked to managing the food context, controlling food shopping, planning meals, having cooking skills, and managing time to prepare and consume meals [7,25], which are factors commonly required for people’s adherence to a GFD. A study to assess eating competence in American women (n = 507) found a positive association between EC and the habit of cooking at home; in addition, the women who were classified as competent eaters reported that they liked cooking and demonstrated more practical skills in managing their meals [53].

Different from EC, the sDOR.2-6y^TM^BR scores did not differ among the participants complying with a GFD. This is probably because parents’ worry about their child staying healthy, showing the clinical challenge of how to follow the sDOR when you have a child who needs lifelong dietary restrictions. A Greek study among 787 healthy children and 141 children with gastrointestinal diseases, aged 2 to 7 years, showed that the diagnosis of a gastrointestinal disease was associated with reduced child autonomy, hampered hunger cues, and the frequent use of distractions during meals [54], which may justify the absence of a relationship between the sDOR and adherence to a GFD in the context of CD. Concerning exclusion diets, the similarity of the food consumed by parents and that consumed by children with a gastrointestinal disease is an important issue, requiring greater knowledge about the adjustment of the eating habits of the family and the adoption of the dietary restrictions in the family context [54,55], which has repercussions on eating dynamics in the family.

The participants whose children complied with a GFD had higher scores for D1 (mealtime structure) but lower scores for D2 (what is available to the child) than those whose children did not comply with a GFD. A GFD requires a better mealtime structure, which may explain the findings in our study. It is worth mentioning that successful compliance with a GFD involves strategies used by families such as planning and taking their own food to social functions [43], which requires adherence to the principles of the D1 domain of the sDOR. In addition to the feeding style, parental and caregiver anxiety about feeding a child with any disorder should also be assessed. Children may be ready to expand their food choices long before the parent feels comfortable doing so [56], which can interfere with what is available to the child, which the D2 domain deals with. The parent or caregiver must trust the child’s instincts on what food is safe and that the child will eat the right quantity of food for them. Children must be able to trust that safe foods will be offered regularly and that they will be allowed to explore new foods in a way that makes them feel comfortable and safe [56]. In our study, income differed only for D3 (how food is available to the child), in which those families up to 2 MW and >10 MW presented the best scores. It is worth noting that gluten-free products are expensive, so income may have interfered with how food was available to the children in question.

In our study, the participants frequently having meals with their family presented the best scores for D1 (mealtime structure) and D3 (how food is available to the child). Previous studies have shown that parents who are competent eaters, eat meals with their children more frequently, and prepare meals together with their children show healthier eating behaviors in addition to evaluating better mealtime structure strategies [8,45]. In other studies, however, children with gastrointestinal disorders have been found to sit at the table during meals or eat the same food as the rest of the family less often compared to children without gastrointestinal disorders [54,57]. The mean score of the sDOR.2-6y^TM^ BR in this study (22.08 ± 3.86) was lower than that in the study performed, in a more general context, among Brazilian parents or caregivers of children between 24 and 72 months of age (23.23 ± 3.67) [21].

The participants whose children had been diagnosed with CD less than one year prior to this study presented higher scores for D5 (who controls what, when, or how much is eaten), and those with children with a CD diagnosis dated more than 3 years prior to the study period showed the best scores for D5. One possible explanation is that a longer time elapsing since disease diagnosis may better educate the child and parents about what is recommended in a GFD, allowing greater autonomy for the children. Furthermore, due to malabsorptive conditions, children with celiac disease may be underweight at the time of diagnosis [10], which may lead to a tendency towards greater parental control over how much they eat. As time passes from diagnosis, weight recovery, and adaptation to a GFD, parents tend to be more relaxed in sharing responsibility with their child regarding how much to eat. Furthermore, parent-reported feeding problems are increased in young children with gastrointestinal diseases, including food neophobia, decreased appetite, prolonged mealtimes, and negotiation overeating, which may be associated with specific aspects of the mealtime environment and parental feeding practices [55,58,59].

In this study, an association was seen for the sDOR.2-6y^TM^BR D1 domain (mealtime structure) and the EC eating attitude, food acceptance, contextual skills, and total domains. These findings corroborate the original sDOR.2-6y^TM^ validation study, which showed that parents with more adherence to the sDOR had higher EC scores [6]. In another US study with mothers (n = 180) of children between 2 and 5 years old, EC was associated with the practice of dividing responsibilities in the children’s diet by determining the times and foods available, allowing the child to decide how much to eat based on their internal signals of hunger and satiety. Furthermore, the mothers with higher EC scores had less restrictive practices regarding their children’s nutrition and supervised more of the items that made up their children’s diet [44].

This study supports the need for greater attention to eating competence and the division of responsibility in feeding in the context of pediatric CD, with improvements in intervention strategies for the individual and the family. Instruments that assess the interactions of parents and children with celiac disease around food are fundamental tools for future research, the formulation of public health policies, intervention evaluations, and the development of care strategies for children with celiac disease and their families.

Our study has limitations, and caution is needed in interpreting and extrapolating this study’s data. The small sample size and the nature of ours being an online study with a self-administered questionnaire bias are evident, as a homogeneous population resulted from the recruitment of participants with a convenience sample, comprising mostly female participants and people with high levels of education and incomes. Also, despite the extensive use of self-reported GFD compliance [19,22,29,30,31,32,33], it was not possible to confirm this information by laboratory tests since data collection occurred online and was spread through all Brazilian regions. The size of the Brazilian territory and the cost of laboratory tests limited the confirmation of GFD compliance.

## 5. Conclusions

This is the first study to evaluate eating competence and the division of responsibility in feeding among Brazilian parents or caregivers of children with celiac disease aged 24–72 months. The participants following a GFD presented higher scores for all EC domains and the total EC. The total EC scores were higher for the participants over 40 y/o, frequently having meals as a family, with children consuming more than three servings of fruit and at least one serving of vegetables daily and complying with a GFD. Different from the EC, the sDOR.2-6y^TM^BR scores did not differ among the participants complying with a GFD. The parents or caregivers of children with CD presented lower scores in the sDOR.2-6y^TM^BR and the ecSI2.0^TM^BR than the study performed, in a more general context, among Brazilian parents or caregivers of children between 24 and 72 months of age. The results showed associations only for the sDOR.2-6y^TM^BR D1 (mealtime structure) and the EC eating attitude, food acceptance, contextual skills, and total domains. Our findings support the need for greater attention to exploring EC and sDOR in the context of pediatric CD, allowing for the development of better forms of care and therapeutic strategies for children with celiac disease and their families.

## Figures and Tables

**Table 1:** Sub-scores and categories of the ecSI2.0^TM^BR scale subcategorized by sociodemographic variables and health and consumption characteristics (n = 50).

	Eating Attitude	Food Acceptance	Internal Regulation	Contextual Skills	Total
	Mean (SD)	Mean (SD)	Mean (SD)	Mean (SD)	Mean (SD)
Age *					
Up to 40 years	11.85 (3.92)	5.00 (2.77)	4.03 (1.63)	9.77 (3.54)	30.64 (9.91)
More than 40 years	14.00 (2.97)	6.09 (1.22)	4.73 (0.90)	11.91 (1.45)	36.73 (4.20)
*p*	0.099	0.066	0.179	0.005	0.005
Schooling level **					
High School	11.00 (5.20)	4.71 (3.30)	3.57 (2.23)	8.86 (5.43)	28.14 (13.42)
Undergraduate	13.88 (3.12)	5.88 (2.31)	4.63 (1.36)	10.94 (2.29)	35.31 (7.47)
Graduate	11.74 (3.64)	5.00 (2.50)	4.07 (1.38)	10.19 (3.16)	31.00 (8.77)
*p*	0.127	0.473	0.275	0.387	0.170
Income ^+^**					
Up to 2 MW	12.2 (5.71)	5.90 (3.00)	3.50 (2.42)	9.30 (5.14)	30.90 (14.46)
3–5 MW	12.5 (3.48)	5.64 (2.10)	4.36 (1.15)	10.50 (2.03)	33.00 (6.91)
6–9 MW	12.00 (3.27)	5.50 (2.51)	4.00 (1.25)	9.40 (3.10)	30.90 (8.75)
More than 10 MW	11.91 (3.56)	4.36 (3.04)	4.18 (1.17)	10.55 (3.39)	31.00 (9.24)
*p*	0.984	0.545	0.594	0.737	0.936
BMI kg/m^2^ *					
Less than 25	12.96 (2.78)	5.32 (2.31)	4.43 (1.14)	10.68 (2.51)	33.39 (6.12)
25 or more	11.60 (4.98)	5.20 (3.00)	3.75 (1.92)	9.55 (4.30)	30.10 (12.73)
*p*	0.232	0.875	0.168	0.301	0.239
Gluten-free diet *					
No	10.79 (3.93)	4.39 (2.78)	3.68 (1.66)	8.75 (3.48)	27.61 (9.55)
Yes	14.27 (2.64)	6.32 (1.73)	4.82 (1.05)	12.14 (1.81)	37.55 (5.11)
*p*	0.001	0.004	0.007	0.000	0.000
Children age *					
2 to 4 years	11.00 (4.51)	4.55 (2.78)	3.65 (1.66)	9.90 (3.73)	29.10 (11.39)
5 to 6 years	13.20 (3.04)	5.70 (2.31)	4.53 (1.33)	10.47 (3.05)	33.90 (7.16)
*p*	0.045	0.118	0.043	0.559	0.073
Children gender *					
Female	12.16 (3.53)	5.28 (2.53)	4.16 (1.53)	9.72 (3.41)	31.31 (8.91)
Male	12.61 (4.37)	5.17 (2.64)	4.22 (1.56)	11.17 (3.00)	33.17 (10.08)
*p*	0.690	0.880	0.885	0.139	0.504
Time since children received CD diagnosis **					
Less than 1 year	12.14 (4.60)	5.23 (2.71)	3.91 (1.74)	9.86 (3.26)	31.14 (10.99)
1 to 3 years	13.24 (3.42)	5.71 (2.05)	4.41 (1.42)	11.12 (2.85)	34.47 (7.96)
More than 3 years	11.27 (2.33)	4.55 (2.94)	4.36 (1.21)	9.64 (4.06)	29.82 (7.08)
*p*	0.403	0.508	0.544	0.406	0.376
Other medical diagnosis *					
No	12.82 (2.61)	4.82 (2.31)	4.29 (1.21)	10.32 (2.86)	32.25 (6.40)
Yes	11.68 (4.94)	5.77 (2.78)	4.05 (1.86)	10.14 (3.88)	31.64 (12.18)
*p*	0.335	0.192	0.585	0.847	0.832
Children with CD complying with a GFD *					
No	9.78 (5.93)	4.22 (3.67)	3.22 (1.79)	7.22 (4.38)	24.44 (14.55)
Yes	12.88 (3.00)	5.46 (2.23)	4.39 (1.39)	10.90 (2.66)	33.63 (6.91)
*p*	0.026	0.353	0.036	0.037	0.006
Frequency of family meals **					
Sometimes	10.50 (4.97)	2.83 (3.06) ^A^	3.83 (1.60)	6.83 (4.07) ^A^	24.00 (12.96) ^A^
Almost always	11.76 (4.07)	4.86 (2.63) ^AB^	3.86 (1.56)	9.33 (3.04) ^AB^	29.81 (9.12) ^AB^
Always	13.30 (3.08)	6.22 (1.81) ^B^	4.57 (1.44)	11.96 (2.29) ^B^	36.04 (6.26) ^B^
*p*	0.190	0.007	0.260	0.000	0.005
Frequency of preparing meals at home *					
Not every day of the week	11.67 (3.60)	4.93 (2.34)	3.80 (1.74)	9.67 (3.02)	30.07 (8.52)
Every day of the week	12.60 (3.92)	5.37 (2.65)	4.34 (1.41)	10.49 (3.44)	32.80 (9.60)
*p*	0.433	0.582	0.252	0.428	0.346
Children’s fruit consumption *					
1 to 2 servings daily	11.69 (3.90)	4.78 (2.56)	3.92 (1.56)	9.72 (3.57)	30.11 (9.66)
3 or more servings daily	13.93 (3.15)	6.43 (2.14)	4.86 (1.23)	11.57 (2.10)	36.79 (6.27)
*p*	0.062	0.038	0.049	0.076	0.021
Children’s vegetables consumption *					
None	9.08 (4.98)	3.00 (2.80)	3.00 (1.86)	7.00 (3.59)	22.08 (11.71)
1 or more servings daily	13.34 (2.72)	5.95 (2.03)	4.55 (1.20)	11.26 (2.49)	35.11 (5.63)
*p*	0.014	0.000	0.001	0.002	0.003
Guided by a dietitian *					
No	12.50 (4.37)	4.91 (2.43)	4.23 (1.90)	10.32 (3.05)	31.95 (9.83)
Yes	12.18 (3.39)	5.50 (2.65)	4.14 (1.18)	10.18 (3.56)	32.00 (9.02)
*p*	0.771	0.421	0.856	0.884	0.987
Participating in celiacs’ group or association *					
No	12.63 (4.00)	4.75 (1.83)	3.75 (1.58)	9.88 (2.47)	31.00 (7.27)
Yes	12.26 (3.83)	5.33 (2.67)	4.26 (1.52)	10.31 (3.47)	32.17 (9.69)
*p*	0.808	0.558	0.389	0.737	0.748
Another person at home with gluten-related disorders *					
No	12.02 (3.78)	5.02 (2.57)	4.11 (1.59)	10.0 (3.35)	31.16 (9.28)
Yes	14.50 (3.67)	6.83 (1.72)	4.67 (0.82)	12.0 (2.53)	38.0 (7.40)
*p*	0.137	0.102	0.409	0.167	0.091

* Student *t*-test. ** Anova with Tukey’s post hoc test (for each variable, groups with the same letters do not differ significantly). ^+^ 1 WS = 1320 1.00 USD = 5.04 BRL (31 October 2023). BMI: body mass index; CD: celiac disease; freq: frequency; GFD: gluten-free diet; MW: minimum wage; and SD: standard deviation.

**Table 2:** Sub-scores and categories of the sDOR.2-6y^TM^BR scales subcategorized by sociodemographic variables and health and consumption characteristics (n = 50).

	D1	D2	D3	D4	D5	Total
	Mean (SD)	Mean (SD)	Mean (SD)	Mean (SD)	Mean (SD)	Mean (SD)
Age *						
Up to 40 years	4.64 (1.42)	2.51 (1.71)	5.82 (1.68)	3.92 (1.98)	5.10 (1.47)	22.00 (3.82)
More than 40 years	5.45 (0.69)	2.09 (1.81)	5.64 (1.75)	4.09 (1.87)	5.09 (1.70)	22.36 (4.18)
*p*	0.012	0.480	0.752	0.803	0.982	0.786
Schooling level **						
High School	4.00 (2.24)	1.71 (1.38)	6.14 (1.07)	3.71 (2.21)	4.86 (2.04)	20.43 (2.15)
Undergraduate	4.94 (1.18)	2.63 (1.86)	5.56 (1.86)	4.00 (1.71)	5.44 (1.31)	22.56 (3.27)
Graduate	4.96 (1.09)	2.48 (1.74)	5.81 (1.73)	4.00 (2.06)	4.96 (1.48)	22.22 (4.47)
*p*	0.218	0.499	0.747	0.939	0.554	0.465
Income ^+^**						
Up to 2 MW	5.10 (1.29)	2.00 (1.41)	6.50 (1.65) ^B^	3.70 (2.26)	5.30 (2.06)	22.60 (3.37)
3–5 MW	4.93 (1.27)	2.14 (1.92)	5.57 (1.55) ^AB^	3.57 (2.06)	5.07 (1.38)	21.29 (2.76)
6–9 MW	4.60 (0.97)	2.80 (1.81)	4.40 (1.71) ^A^	4.40 (1.17)	5.10 (1.20)	21.30 (2.26)
More than 10 MW	4.82 (1.17)	2.82 (1.60)	6.64 (0.92) ^B^	4.36 (2.01)	5.36 (1.43)	24.00 (4.34)
*p*	0.814	0.569	0.005	0.634	0.958	0.165
BMI kg/m^2^ *						
Less than 25	4.71 (1.41)	2.61 (1.81)	5.68 (1.74)	4.32 (1.81)	5.25 (1.46)	22.57 (4.37)
25 or more	4.90 (1.29)	2.35 (1.60)	5.75 (1.62)	3.85 (1.79)	4.95 (1.64)	21.80 (3.02)
*p*	0.644	0.614	0.886	0.375	0.507	0.499
Gluten-free diet *						
No	4.50 (1.26)	2.82 (1.76)	5.61 (1.66)	3.89 (1.87)	5.11 (1.47)	21.93 (3.86)
Yes	5.23 (1.34)	1.91 (1.57)	6.00 (1.72)	4.05 (2.06)	5.09 (1.57)	22.27 (3.94)
*p*	0.055	0.063	0.418	0.785	0.970	0.758
Children age *						
2 to 4 years	4.95 (1.19)	2.60 (1.39)	5.30 (1.53)	4.05 (2.11)	5.25 (1.83)	22.15 (4.33)
5 to 6 years	4.73 (1.44)	2.30 (1.93)	6.10 (1.73)	3.90 (1.84)	5.00 (1.26)	22.03 (3.59)
*p*	0.579	0.553	0.100	0.792	0.598	0.918
Children gender *						
Female	4.91 (1.25)	2.56 (1.79)	6.03 (1.67)	4.09 (1.91)	4.94 (1.22)	22.53 (3.76)
Male	4.67 (1.50)	2.17 (1.62)	5.33 (1.64)	3.72 (2.02)	5.39 (1.91)	21.28 (4.01)
*p*	0.548	0.442	0.161	0.521	0.375	0.275
Time since children received CD diagnosis **						
Less than 1 year	4.91 (1.31)	2.68 (1.70)	5.68 (1.64) ^AB^	4.00 (1.66)	5.77 (1.60) ^B^	23.05 (3.57)
1 to 3 years	5.12 (0.93)	1.82 (1.59)	5.24 (1.86) ^A^	3.76 (2.14)	4.59 (1.42) ^AB^	20.53 (4.03)
More than 3 years	4.18 (1.78)	2.82 (1.89)	6.82 (0.98) ^B^	4.18 (2.27)	4.55 (0.82) ^A^	22.55 (3.72)
*p*	0.179	0.213	0.045	0.855	0.016	0.117
Other medical diagnosis *						
No	4.86 (1.38)	2.43 (1.55)	5.86 (1.60)	4.07 (2.16)	4.96 (1.60)	22.18 (4.61)
Yes	4.77 (1.31)	2.41 (1.97)	5.68 (1.81)	3.82 (1.65)	5.27 (1.39)	21.95 (2.72)
*p*	0.827	0.969	0.718	0.651	0.477	0.841
Children with CD complying with a GFD *						
No	3.44 (1.24)	3.67 (1.50)	5.00 (1.12)	4.67 (1.12)	5.33 (1.87)	22.11 (1.69)
Yes	5.12 (1.17)	2.15 (1.67)	5.95 (1.75)	3.80 (2.05)	5.05 (1.43)	22.07 (4.20)
*p*	0.000	0.015	0.126	0.231	0.612	0.979
Frequency of family meals **						
Sometimes	3.83 (1.17) ^A^	3.00 (1.67)	5.50 (1.64) ^AB^	4.50 (1.05)	5.50 (1.05)	22.33 (2.80)
Almost always	4.38 (1.12) ^B^	2.90 (1.64)	5.05 (1.40) ^A^	4.19 (1.78)	5.10 (1.73)	21.62 (3.89)
Always	5.48 (1.27) ^B^	1.83 (1.70)	6.52 (1.68) ^B^	3.61 (2.23)	5.00 (1.41)	22.43 (4.15)
*p*	0.002	0.078	0.011	0.477	0.775	0.778
Frequency of preparing meals at home *						
Not every day of the week	4.33 (1.72)	3.13 (1.88)	5.20 (1.86)	4.07 (1.44)	5.07 (1.16)	21.80 (2.76)
Every day of the week	5.03 (1.10)	2.11 (1.59)	6.03 (1.56)	3.91 (2.13)	5.11 (1.64)	22.20 (4.28)
*p*	0.092	0.055	0.111	0.802	0.919	0.741
Children’s fruit consumption *						
Up to 2 servings daily	4.78 (1.12)	2.44 (1.58)	5.56 (1.58)	4.08 (1.84)	5.06 (1.53)	21.92 (3.70)
3 or more servings daily	4.93 (1.82)	2.36 (2.13)	6.36 (1.86)	3.64 (2.21)	5.21 (1.48)	22.50 (4.36)
*p*	0.724	0.874	0.132	0.476	0.741	0.936
Children’s vegetables consumption *						
None	4.33 (1.30)	2.42 (1.73)	5.92 (1.68)	3.50 (1.98)	5.75 (1.36)	21.92 (3.75)
1 or more servings daily	4.97 (1.33)	2.42 (1.75)	5.74 (1.70)	4.11 (1.93)	4.89 (1.50)	22.13 (3.94)
*p*	0.149	0.994	0.750	0.351	0.085	0.868
Guided by a dietitian *						
No	4.91 (1.54)	2.23 (1.77)	6.14 (1.70)	3.82 (2.13)	5.18 (1.53)	22.27 (3.84)
Yes	4.75 (1.17)	2.57 (1.71)	5.50 (1.64)	4.07 (1.80)	5.04 (1.50)	21.93 (3.93)
*p*	0.680	0.490	0.187	0.651	0.737	0.758
Participating in celiacs’ group or association *						
No	5.38 (0.92)	1.50 (2.27)	6.75 (1.67)	2.75 (2.25)	5.13 (0.99)	21.50 (5.15)
Yes	4.71 (1.38)	2.60 (1.58)	5.60 (1.64)	4.19 (1.81)	5.10 (1.59)	22.19 (3.63)
*p*	0.203	0.101	0.075	0.053	0.960	0.648
Another person at home with gluten-related disorders *						
No	4.73 (1.37)	2.48 (1.72)	5.86 (1.68)	3.98 (1.97)	5.16 (1.54)	22.20 (3.97)
Yes	5.50 (0.84)	2.00 (1.90)	5.17 (1.72)	3.83 (1.83)	4.67 (1.21)	21.17 (3.06)
*p*	0.186	0.531	0.346	0.866	0.457	0.542

* Student *t*-test. ** Anova with Tukey’s post hoc test (for each variable, groups with the same letters do not differ significantly). ^+^ 1 WS = 1.320,00 1.00 USD = 5.04 BRL (31 October 2023). D1—Mealtime structure. D2—What is available to the child. D3—How food is available to the child. D4—Respect for child’s autonomy in eating. D5—Who controls what, when, and how much is eaten. BMI: body mass index; CD: celiac disease; freq: frequency; GFD: gluten-free diet; MW: minimum wage; and SD: standard deviation.

**Table 3:** Associations between sDOR.2-6y^TM^BR and ecSI2.0^TM^BR scores (and its domains) (n = 50).

			ecSI2.0^TM^BR		
sDOR.2-6y^TM^	Eating Attitude Pearson’s Correlation (p)	Food Acceptance Pearson’s Correlation (p)	Internal Regulation Pearson’s Correlation (p)	Contextual Skills Pearson’s Correlation (p)	Total Pearson’s Correlation (p)
D1	0.344 (0.014)	0.470 (0.001)	0.177 (0.219)	0.448 (0.001)	0.459 (0.001)
D2	−0.151 (0.295)	−0.214 (0.136)	−0.224 (0.119)	−0.196 (0.172)	−0.227 (0.113)
D3	0.075 (0.606)	0.084 (0.561)	0.072 (0.621)	0.105 (0.468)	0.103 (0.477)
D4	0.060 (0.680)	−0.073 (0.617)	−0.088 (0.546)	−0.145 (0.316)	−0.061 (0.672)
D5	−0.056 (0.701)	−0.108 (0.456)	−0.008 (0.956)	−0.046 (0.751)	−0.070 (0.629)
Total	0.093 (0.523)	0.025 (0.863)	−0.055 (0.706)	0.022 (0.877)	0.044 (0.762)

D1—Mealtime structure. D2—What is available to the child. D3—How food is available to the child. D4—Respect for child’s autonomy in eating. D5—Who controls what, when, and how much is eaten.

## Data Availability

Data are contained within the article and Supplementary Materials.

## Supplementary Materials

The following supporting information can be downloaded at https://www.mdpi.com/article/10.3390/nu16071052/s1: Table S1: Sociodemographic and health and consumption characteristics (n = 50).
