# Supplementary material for: Division of Responsibility in Child Feeding and Eating Competence: A Cross-Sectional Study in a Sample of Caregivers of Brazilian Children with Celiac Disease
Source: Nutrients. 2024 Apr 4;16(7):1052. doi: 10.3390/nu16071052 (PMC11013579; doi:10.3390/nu16071052)
Supplement: Supplementary file 1 [file nutrients-16-01052-s001.zip › nutrients-2888680-supplementary.pdf]

Table S1. Socio-demographic and health and consumption characteristics (n=50).

		Sample (N =50)	
		**freq**	**%**
**Gender**	Female	49	98.0%
	Male	1	2.0%
**Age**	Up to 40 years	39	78.0%
	More than 40 years	11	22.0%
**Schooling level**	High School	7	14.0%
	Undergraduate	16	32.0%
	Graduate	27	54.0%
**Income**	Up to 2 MW	10	20.0%
	3 – 5 MW	14	28.0%
	6 – 9 MW	10	20.0%
	More than 10 MW	11	22.0%.
	Prefer not to inform	5	10.0%
**BMI kg/m²**	Low: <18.5	2	4.2%
	Normal: 18.5–24.9	26	54.2%
	Overweight: 25.0–29.9	12	25.0%
	Obesity: ≥ 30	8	16.7%
	Missing	2	4.0%
**Gluten free diet**	No	28	56.0%
	Yes	22	44.0%
**Children age**	2 to 4 years	20	40.0%
	5 to 6 years	30	60.0%
**Children gender**	Female	32	64.0%
	Male	18	36.0%
**Time since CD children diagnosis**	Less than 1 year	22	44.0%
	1 to 3 years	17	37.0%
	More than 3 years	11	22.0%
**Other medical diagnosis**	No	28	56.0%
	Yes	22	44.0%
**CD children complying with GFD**	No	9	18.0%
	Yes	41	82.0%
**Frequency of family meals**	Sometimes	6	12.0%
	Almost always	21	42.0%
	Always	23	46.0%
**Frequency of preparing meals at home**	1 to 3 times per week	2	4.0%
	4 to 6 times per week	13	26.0%
	7 days per week	35	70.0%

**Children's fruits consumption**	None	2	4.0%
	1 to 2 portions daily	34	68.0%
	3 to 4 portions daily	12	24.0%
	5 ou more portions daily	2	4.0%
**Children's vegetables consumption**	None	12	24.0%
	1 to 2 portions daily	36	72.0%
	3 to 4 portions daily	2	4.0%
**Guided by a dietitian**	No	22	44.0%
	Yes	28	56.0%
**Participating in celiacs'groups or associations**	No	8	16.0%
	Yes	42	84.0%
**Another person at home with gluten-related disorders**	No	44	88.0%
	Yes	6	12.0%

BMI: body mass index
CD: celiac disease
GFD: gluten-free diet
